# The role of phosphodiesterase-5 inhibitors in prostatic inflammation: a review

**DOI:** 10.1186/s12950-015-0099-7

**Published:** 2015-09-15

**Authors:** Christina Alves Peixoto, Fabiana Oliveira dos Santos Gomes

**Affiliations:** Laboratório de Ultraestrutura, Centro de Pesquisas Aggeu Magalhães (CPqAM-FIOCRUZ), Fundação Oswaldo Cruz, Av. Moraes Rego s/n, CEP: 50670-420, Cidade Universitária, Recife, PE Brazil

**Keywords:** Phosphodiesterase-5 inhibitors, BPH, LUTS, Inflammation

## Abstract

Clinical and basic experimental evidence indicates that chronic inflammation is the greatest factor in benign prostatic hyperplasia (BPH) progression, which is the most common cause of Lower Urinary Tract Symptoms (LUTS). The use of anti-inflammatory agents such as steroids, cyclooxygenase-2 (COX-2) and phytotherapics have been investigated as forms of treatment for various prostate diseases. Recent evidence has demonstrated that PDE5 inhibitors (PDE5Is) improve symptoms of BPH/LUTS, possibly as a result of the relaxing of the smooth muscle fibers of the bladder and prostate by NO/cGMPc signaling, or by improving RhoA/Rho-kinase (ROCK), and reduction of the hyperactivity of the autonomic nervous system. However, some results have suggested that besides vasodilatation and their anti-proliferative effect, PDE5Is exert a direct anti-inflammatory effect, by raising cGMP. Given that inflammation is major factor in benign prostatic hyperplasia (BPH) progression, PDE5Is could act also restore prostatic function as they act as potent anti-inflammatory drugs. This review aims to provide a comprehensive summary of the use of phosphodiesterase-5 inhibitors to treat prostatic inflammation.

## Introduction

The cyclic monophosphate nucleotides cAMP and cGMP are synthesized by the guanylyl and adenylyl cyclase enzymes, respectively. They are recognized as important second messengers of extracellular signals. Through cyclic dependent protein kinases, PKA and PKG, control physiological functions, such as retinal phototransduction, smooth muscle relaxation, cardiac contractility, neuroendocrine signals, inhibition of platelet aggregation, degranulation of neutrophils, and myelogenic inflammatory response [1–5]. The critical role of cAMP and cGMP in intracellular signaling pathways has identified them as potential therapeutic targets [6, 7].

Mammalian phosphodiesterases (PDEs) comprise a large group of enzymes that hydrolyze cAMP and cGMP to their inactive forms 5′-GMP and 5′-AMP [8, 9]. Eleven PDE families have been described in order of discovery, amino acid sequence and catalytic and regulatory characteristics. Some PDEs, such as PDE4 and PDE7 are highly specific for cAMP hydrolysis, whereas PDE5, PDE6 and PDE9 are specific for cGMP. Some PDEs, such as PDE1, PDE2 and PDE3 hydrolyze both nucleotides [10]. Therefore, inhibitors of PDE (PDEIs) can prolong the intracellular action of cAMP and cGMP. There are potent non-selective PDEi, such as theophiline, and others that are specific, such as Rolipram for PDE4, and Sildenafil, Tadalafil and Vardenafil for PDE5 [7].

Potent and selective PDE5 inhibitors have been approved for therapeutic use for the condition of erectile dysfunction [11] and are currently also being used in the treatment of pulmonary hypertension [12, 13] and Raynaud’s phenomenon [14].

cGMP accumulation may inhibit inflammation, and as such it is a potential tool against the evolution of diseases in which inflammation plays a central role [15, 16, 8, 17–19].

Chronic prostatitis is characterized by pelvic and genitourinary pain and lower urinary tract symptoms (LUTS) that affect frequency and urgency and causes dysuria. Its precise aethiology remains unclear, although some possibilities include undetectable infection, trauma and immunological origin. Therapeutic management includes antibiotics and α-adrenergic blockers, but until now, no definitive treatment has been identified [20].

In this way, phosphodiesterase-5 inhibitors may not only mediate smooth muscle relaxation, but also can directly reduce prostatic inflammation by increasing cGMP levels.

## Phosphodiesterase-5 and inflammation

The nitric oxide (NO) is a highly reactive molecule with diverse physiological functions [21]. This messenger plays important roles in the modulation of vascular tone [22], neurotransmission [23, 24] and the immune system [25, 26]. NO is formed from L-arginine by NO synthases (NOS). In addition to the constitutive forms of the enzyme, endothelial (eNOS or NOS3) and neuronal (nNOS ou NOS1), the inducible form (iNOS or NOS2) is found in activated macrophages and other immune cells that produce NO in the micromolar range [27]. At these concentrations, NO induced oxidative DNA damage and modified protein structure and function, which can lead to cell death. Controversially, eNOS produces NO at nanomolar concentrations, which have been documented to have anti-inflammatory actions [28], which appear to be related directly or indirectly to the inhibition of the key transcription factor Nuclear Factor κB (NF-κB) [29, 30].

NO is also the main activator of soluble guanylyl cyclase (sGC), an enzyme that synthesizes cGMP. The level of cGMP is regulated by phosphodiesterases type 5 (PDE5s) enzymes, which break down the phosphodiesteric bond of cGMP. Several PDE5 inhibitors (PDE5Is) have been developed and used as therapeutic agents, as they increase cyclic nucleotide levels by blocking PDE function, enhancing NO-cGMP signalization [7].

Current concepts based on clinical and experimental data support a link between endothelial dysfunction and inflammation, manifested as deficiencies in the production of NO and prostacyclin [31]. The chronic consequence of endothelial dysfunction is initiation of vascular diseases. Sildenafil, the selective PDE5 inhibitor, widely used for treatment of erectile dysfunction in humans (Viagra®, Pfizer) has been shown to improve NOS activation of endothelial cells through ERK signaling [32]. In addition to increasing NO production by eNOS activation, Sildenafil also reduces the oxidative stress induced by hyperglicaemia and insulin resistance conditions [33]. Sildenafil also stimulates eNOS mRNA transcription in cardiomyocytes, resulting in increased expression of eNOS, elevated NO generation, guanylyl cyclase activation and enhanced GMPc formation [34]. Therefore, Sildenafil can elevate cGMP in two alternative ways: inhibiting PDE5 enzymes and/or inducing mRNA expression of eNOS.

Interestingly, acute and short-term administration of Sildenafil improves endothelial function in men with Type 2 diabetes [35], whereas chronic administration of Sildenafil, besides significantly improving endothelial function, can also reduce inflammatory markers (nitrite/nitrate levels, C-reactive protein, IL-6, ICAM-1 and VCAM-1) in patients with Type 2 diabetes [36]. In addition to improving endothelial function in patients with coronary arterial disease and diabetes, and reducing oxidative stress in many tissues [37, 38], Sildenafil can also normalize endothelial function and decrease plaque deposition in the aorta in experimental models of atherosclerosis [39].

Sildenafil has also been shown to be of potential benefit in the early phases of inflammation and vascular remodeling in a pulmonary arterial hypertension (PAH) experimental model. The administration of Sildenafil following Monocrotaline-induced PAH significantly reduced the severity of inflammation in the acute stage of the disease and prevented pulmonary arterial remodeling. These results suggest that in addition to its vasodilatation and anti-proliferative effects, Sildenafil has a direct anti-inflammatory effect [40].

The beneficial effect of the phosphodiesterase-5 inhibitor has been demonstrated in experimental inflammatory bowel disease (IBD), a relapsing and remitting disease appearing as a form of ulcerative colitis or Crohn’s disease with a non-well-known etiology. Treatment of experimental IBD with Sildenafil reduced markers of oxidative stress such as myeloperoxidase (MPO) and lipid peroxidation product (TBARS) significantly, thereby modulating the inflammatory response [18]. Recent studies extended and confirmed the anti-inflammatory effect of PDE5 inhibitor in a rat model of colitis [41]. Other inhibitors of the PDE superfamily have been also proposed for IBD treatment, and PDE4, PDE5 and PDE7 inhibitors seem strong candidates for the next generation of effective drugs [42].

In caecal ligation and puncture (CLP), a model of polymicrobial sepis, PDE5 markedly attenuated injury in vital organs such as the kidney and lungs by inhibiting proinflammatory cytokine response and ROS generation [43].

Increased intracellular cGMP levels also lead to the suppression of colon tumor cell growth and the induction of apoptosis by activating cGMP dependent protein kinase (PKG). The COX inhibitory metabolite of sulindac, sulindac sulfide, as well as several other NSAIDs, such as indomethacin, meclofenamic acid and celecoxib, also inhibit PDE5 activity, which is closely associated to tumor cell growth [44, 45]. Studies involving human clinical specimens which reported higher PDE5 levels in colorectal, bladder, lung, and breast carcinomas than with normal epithelium [46–49] corroborate these results.

Myeloperoxidase (MPO) level in the blood can be considered as a marker of endothelial dysfunction and could be a predictor of cardiovascular disease risk. The oxidation of LDL by MPO (MoxLDL) leads to a specific induction of the inflammatory response, increasing the release of cytokines such as interleukin 8 (IL-8) and tumor necrosis factor alpha (TNF-α) by endothelial cells and monocytes respectively [50]. According to Roumeguère et al [51] among the three available specific PDE5-Is for treatment of erectile dysfunction (ED), only tadalafil decreased the inflammatory response on endothelial cells stimulated by myeloperoxidase-modified low-density lipoprotein (Mox-LDLs) or tumor necrosis factor alpha. Mox-LDLs have been found in the atherosclerotic plaque but also in the corpus cavernosum of patients with ED of vascular origin [51].

Varma et al., [52] proposed that PDE5is have cardioprotective effects and also could be used to treat insulin resistance and inflammation. These authors provided evidence that tadalafil therapy reduced circulating inflammatory cytokines in diabetic animal model while improved fasting glucose levels and reduced infarct size after ischemia-reperfusion injury in the heart [52].

cAMP and cGMP may play a protective role in the modulation of some inflammatory cell activities of allergic disorders. Sildenafil inhibits inflammation and airway reactivity in animal models of airway diseases (asthma and chronic obstructive pulmonary disease), the effectiveness of which does not appear to be dependent on endogenous NO levels [53]. Similarly, vardenafil mimics the effect of NO by increasing GMPc levels with a subsequent reduction of histamine release and mast-cell-mediated allergic reactions [54]. Other studies reported that selective PDE4 inhibitors suppressed hematological and immunological markers of inflammation and were also effective in reducing specific airway resistance [55, 56].

The PDE5 inhibitors have also muscle and neuroprotective activities. The administration of sildenafil after bilateral cavernosal nerve resection preserves penile corporal smooth muscle and ameliorates fibrotic degeneration by down-regulating genes related to fibrosis and up-regulating genes related to smooth muscle preservation [57]. At the level of the pelvic ganglia, sildenafil exerts a neuroprotective effect by activating neurotrophic factors involved in neuronal survival and regeneration [58].

Garcia et al., [59] demonstrated that sildenafil can attenuate inflammation and oxidative stress in damaged cavernosal nerves by modulating cytokine expression and promoting a neuroprotective environment that favors neuron survival. According to such authors, initiation of the treatment immediately after surgery or even before radical prostatectomy would produce a better outcome, by promoting regeneration and functional recovery of the peripheral nerves [59].

Other studies have shown that the selective PDE5 inhibitors raise cGMP levels in the brain and offer protective effects, such as improvement of cognition and memory [60], reduction of neuronal cell death in ischemic cerebrovascular injury [61], reduction of white matter damage and regulation of inflammatory responses in Multiple Sclerosis models [62]. Interestingly, Alzheimer’s disease (AD) has been highly associated with cGMP signaling dysfunction and an ongoing inflammatory process. Zhang et al., [63] demonstrated that sildenafil prevents neuroinflammation, lowers beta-amyloid levels and improves cognitive performance in APP/PS1 transgenic mice, an AD experimental model [63]. On the other hand, Garcia-Barroso showed that tadalafil cross the blood-brain barrier and inhibits PDE5 present in the hippocampus, and that tadalafil was more effective than sildenafil in attenuating the phenotypic traits of a mouse model of AD [64].

Results obtained in our laboratory using a multiple sclerosis (MS) model demonstrated that sildenafil exerts an effective anti-inflammatory action, greatly reducing levels of IFN-γ, TNF-α, IL-1β, IL-2 and cycloxygenase-2 (COX-2), as well as protecting myelin structure. Therefore, the oral administration of sildenafil can be a possible therapeutic tool for individuals with MS and other neuroinflammatory/neurodegenerative diseases, providing additional benefits to those of current treatments [4].

## Phosphodiesterases and prostatic inflammation

Benign prostatic hyperplasia (BPH) is a common cause of Lower Urinary Tract Symptoms (LUTS), which include poor urinary stream, urinary hesitancy, a feeling of incomplete bladder emptying, urgent and/or frequent urination, and urge incontinence. Approximately 40 % of men will have BPH by the age of 50, and 80 % by the age of 80 [65].

Clinical and basic experimental evidence indicates that chronic inflammation is the major factor in benign prostatic hyperplasia (BPH) progression. Recognition of prostate secretion products by autoreactive T cells and animal models on experimental prostatitis demonstrate an autoimmune component to chronic inflammation. The close association between activated T cells and stromal cells suggests that these T cells trigger abnormal growth in the prostate as a result of their specific cytokine production. In BPH, these T cell-derived growth factors are strongly up-regulated and have been documented as stimulating stromal growth, matrix formation and angiogenesis [66]. Consistent with these findings, the REDUCE clinical trial has shown a relationship between prostatic inflammation and prostate volume, and the severity of LUTS [67].

A link between inflammation and prostate proliferative diseases such as BPH and Prostate Cancer (PCa) has also been suggested. Inflammatory infiltration is present in approximately 40 % of cases of patients with BPH, who have a significantly higher risk of BPH progression and acute urinary retention. Evidence from genetic studies supports the hypothesis that prostate inflammation may be a cause of PCa development [68].

In several types of human carcinoma, such as colon adenocarcinoma, bladder squamous carcinoma and lung cancers, PDE-5 expression is elevated, suggesting that these enzymes play a role in controlling cellular proliferation and apoptosis mechanisms [49, 69]. Additionally, in human prostate cancer cell lines, studies suggested that the increase of cAMP and cGMP initiates morphologic differentiation, inhibiting the growth and the invasive potential of these cells [70, 71].

Anti-inflammatory agents, whose effects are promising in terms of inhibition of cell proliferation [72], have been analyzed for the treatment of various prostate diseases such as steroids, cyclooxygenase-2 (COX-2) and phytotherapics. *In vitro* studies also found evidence of the antiproliferative effect of PDE inhibitors in smooth muscle cells from human BPH tissue [73, 74].

Preclinical and clinical studies have provided evidence that PDE5 inhibitors improve symptoms of Benign Prostatic Hyperplasia/Symptoms of Upper Urinary Tract (BPH/LUTS), possibly as a result of their relaxing action via NO mechanisms, and inhibition of prostatic stromal cells proliferation [75–77]. The possible use of PDE5 inhibitors for the treatment of prostate diseases is supported by the presence of PDE5 in the transition zone of the prostate, together with PDE4 and PDE11 [8], as well as the presence of PDE5 in blood vessels and in the muscular fibers of the bladder and urethra [78].

Several randomized, double-blind, placebo-controlled, multinational trials have investigated the efficacy and safety of tadalafil [79–87] or sildenafil [88, 89, 79, 90–92] in the treatment of BPH-LUTS, as well as in the treatment of men with ED and with BPH-LUTS, leading to regulatory approval in the USA and Europe.

Nonsystematic and systematic reviews have tried to analyze the role of combined PDE5Is and α-blocker therapy, and have reported a significant improvement in urinary symptoms [76, 92–95]. The most remarkable outcome from the first systematic review was that the combination of PDE5Is and α-adrenergic blockers can significantly improve maximum urinary flow rate, compared with only α-adrenergic blockers, whereas PDE5Is only did not increase Qmax, compared with placebo [92].

Similarly, a recent systematic review and network meta-analysis comparing the effectiveness of oral drug therapies for BPH/LUTS revealed that of all the available drug treatments, combination therapy with α1-adrenoceptor antagonists and PDE5 inhibitor ranked highest in efficacy for decreasing the International Prostate Symptom Score (IPSS) total score, storage subscore and voiding subscore. PDE5 inhibitors used alone also had a promising effect, except on maximum flow rate (Qmax). The results suggested that this combination therapy is the most efficient treatment of LUTS/BPH [96].

In 2010, Eryildirim et al. evaluated the effectiveness of sildenafil citrate on lower urinary system symptoms (LUTS) by using symptom score scales, and by analyzing whether or not the presence of asymptomatic inflammatory prostatitis altered the symptom scores. Patients were classified as category IV prostatitis (asymptomatic inflammatory prostatitis) by the presence of significant leukocytes (or bacteria or both) in secretion extracted by prostate massage and urine obtained after the massage. In cases of LUTS and ED without asymptomatic inflammatory, sildenafil citrate had an improving effect on LUTS as well as on ED. However, in cases with asymptomatic inflammatory prostatitis, sildenafil citrate did not lead to an improvement in LUTS [88]. In addition to the limitation of the study, which did not include a placebo group, was not randomized, and had a small sample size, the absence of results could be explained by the low number of PDE5Is doses, which were restricted to 50 mg sildenafil citrate administered twice a week for 30 days, ideal for ED treatment but not for chronic inflammation therapy.

Grimsley et al., proposed a hypothesis to explain the mechanism of action of sildenafil when ameliorating prostatitis symptoms. According to the authors these effects can be explained by the relaxation of the prostatic duct smooth muscle increasing washout of prostatic reflux products [20].

Cantoro et al. [89] evaluated the effectiveness of tamsulosin (α-adrenergic blocker) monotherapy versus tamsulosin plus sildenafil combination therapy on erectile dysfunction (ED) in young patients with type III chronic prostatitis, by using symptom score scales. They observed that tamsulosin monotherapy, as well as a combination therapy (tamsulosin plus sildenafil) had an improving effect on symptoms and on ED in patients with type III prostatitis [89].

Whether PDE5Is an effective prostatitis treatment or not remains controversial. However, it is important to highlight that until today pre-clinical and clinical studies have featured doses and short-term treatment, ideal for ED and BPH/LUTS treatment, not for chronic inflammation therapy. Although several experimental and clinical studies have found evidence of their possible benefits, no chronic treatment with PDE5Is has been performed to evaluate their effects on the human prostatitis.

It is important also to point out that PDE5Is have been chronically used as a pharmacological strategy for several non-urological disorders, such as pulmonary hypertension, Raynaud’s phenomenon and altitude sickness [76]. Although PDE5Is are considered safe drugs with few side effects, long-term studies are needed to evaluate their effects on the normal male reproductive system, specifically on the prostate. The ultrastructural and molecular analysis realized by our group demonstrated that chronic treatment of C57Bl/6 mice with sildenafil 25 mg/kg for 4 weeks enhanced prostatic glandular activity, however, no differences were observed in sGC, eNOS, PSA and TGF-β expression [97]. These results suggested that the chronic use of sildenafil does not cause evident prostatic damage, and therefore, seems safe for the treatment of a variety of disorders.

Recent studies have demonstrated that BPH/LUTS, prostatic cancer and metabolic syndrome (MetS) are often associated with one another [98]. Metabolic syndrome (MetS) is a complex of clustering metabolic abnormalities and comprises a number of disorders such as insulin resistance, hypertension and obesity, which all act as known risk factors for erection dysfunction (ED).

Interestingly, some studies have shown that treatment with PDE5Is, in addition to relaxing the muscular wall, may positively affect low urinary tract blood perfusion, restoring function and causing morphologic changes in the bladder and prostate induced by chronic pelvic ischemia caused by MetS or hypertension [99, 100]. In spontaneous hypertensive rats, chronic treatment with tadalafil or other PDE5Is counteracted all LUTS alterations, most likely through increased blood perfusion and oxygenation [99–101].

Hypertension, obesity, and hyperinsulinaemia have all been associated with increased sympathetic activity via enhanced glucose metabolism. This process promotes the activity of α-adrenergic receptors, increasing the smooth muscle tone of the male genitourinary tract [102, 103]. Insulin-like growth factor-1 (IGF-1) contributes to the development and progression of BPH/LUTS. Since these molecules have a similar structure, insulin can bind to IGF-1 receptors and activate the signaling pathway for the growth and proliferation of epithelial and stromal prostatic cells [104]. Therefore, PDE5Is could be used as pharmacological tools for the treatment of ED, LUTS/BPH and chronic pelvic ischemia by smooth muscle relaxation via cGMP-dependent RhoA/Rho-kinase (ROCK) signaling inhibition [105–108], and possibly by reducing autonomic hyperactivity, which is a component of the metabolic syndrome [109].

Moreover, chronic inflammation has also been claimed to be one of the putative links between MetS and BPH/LUTS. Recently, Vignozzi et al. [110] demonstrated that the PDE5 blockade exerts anti-inflammatory effects on human myofibroblast prostatic cells, blunting inflammatory and metabolic insults. These authors showed that treatment with tadalafil or vardenafil suppressed IL-8 and IP-10 secretion induced by inflammatory (TNF-α) and metabolic (oxLDL, AGE and IGF-1) stimuli. PDE5Is also inhibited the expression of inflammatory, fibroblast-to-myofibroblast transdifferentiation and tissue remodelling marker genes, most likely via the activation of cGMP/PKG signaling [110].

## Conclusion

**Fig. 1:**
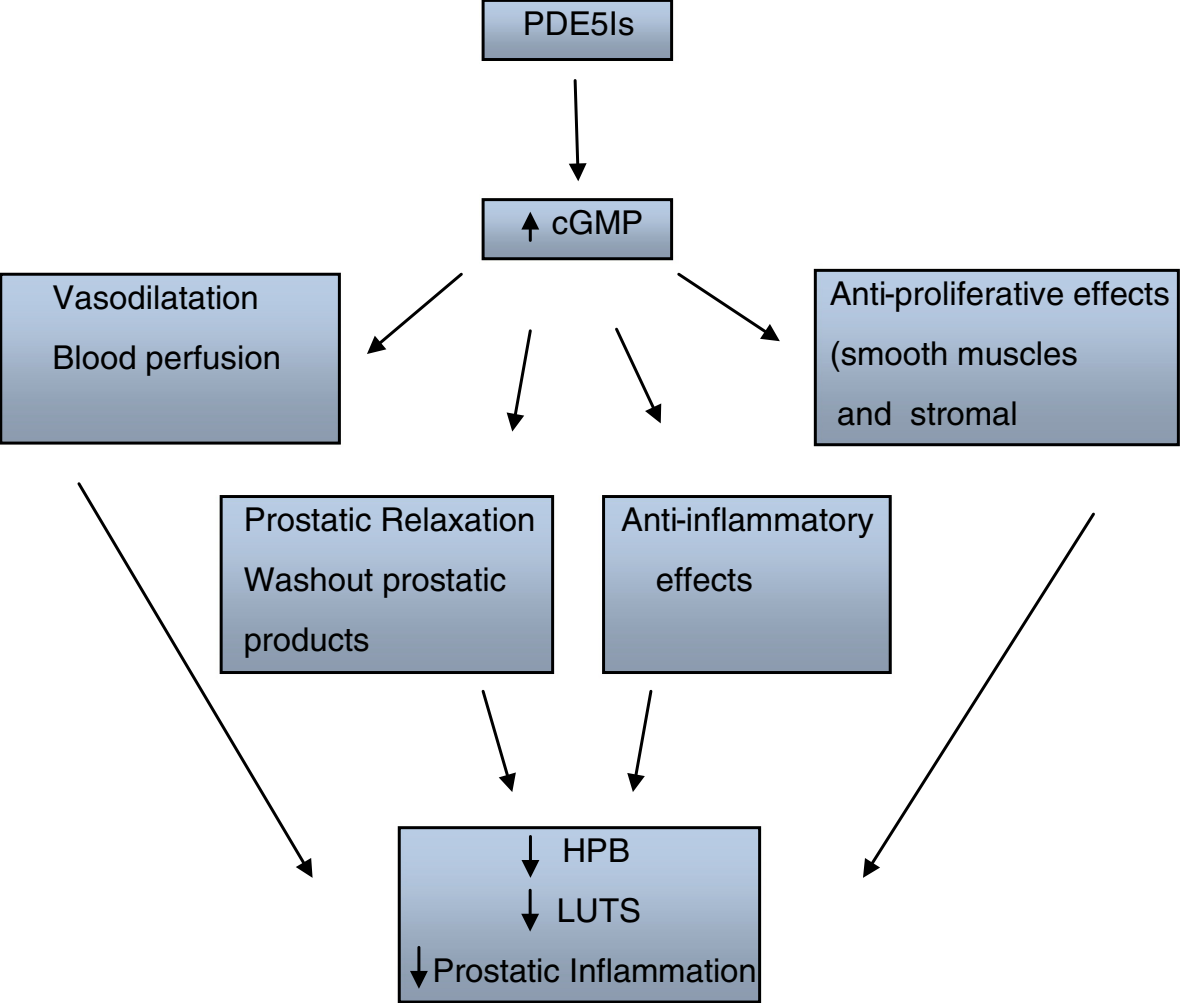
Schematic diagram showing the hypothetical mechanism of Phosphodiesterase 5 Inhibitors (PDE5Is) on prostatic inflammmation. PDE5Is can direct and indirectly down-regulate prostatic inflammation/BPH/LUTS by inducing high levels of cGMP

PDE5Is are therapeutical tools used for several urological and non-urological disorders, and experimental evidence suggest that their chronic use does not induce cellular and molecular prostatic alterations. The mechanisms involved in improvements observed in BPH/LUTS possibly include relaxation of the smooth muscles of the bladder and prostate by NO/cGMPc signaling or via improving RhoA/Rho-kinase (ROCK), and by reduction of the hyperactivity of the autonomic nervous system. PDE5Is can also direct and indirectly down-regulate prostatic inflammation/BPH/LUTS by inducing high levels of cGMP (Fig. 1). In conclusion, since inflammation is a major factor in benign prostatic hyperplasia (BPH) progression, PDE5Is could also restore prostatic function, as they act as potent anti-inflammatory drugs.
